# Glucose chemical exchange saturation transfer MRI for predicting the histological grade of rectal cancer: a comparative study with amide proton transfer-weighted and diffusion-weighted imaging

**DOI:** 10.1186/s13244-024-01828-z

**Published:** 2024-11-11

**Authors:** Nan Meng, Zhun Huang, Han Jiang, Bo Dai, Lei Shen, Xue Liu, Yaping Wu, Xuan Yu, Fangfang Fu, Zheng Li, Zhiwei Shen, Baiyan Jiang, Meiyun Wang

**Affiliations:** 1 Department of Radiology, Henan Provincial People’s Hospital & Zhengzhou University People’s Hospital, Zhengzhou, China; 2 Laboratory of Brain Science and Brain-Like Intelligence Technology, Institute for Integrated Medical Science and Engineering, Henan Academy of Sciences, Zhengzhou, China; 3 Biomedical Research Institute, Henan Academy of Sciences, Zhengzhou, China; 4 Department of Radiology, Xinxiang Medical University Henan Provincial People’s Hospital, Zhengzhou, China; 5 Philips Healthcare, Beijing, China

**Keywords:** Rectal neoplasms, Neoplasm grading, Glucose, Magnetization transfer contrast imaging, Diffusion magnetic resonance imaging

## Abstract

**Background:**

To evaluate the utility of glucose chemical exchange saturation transfer (glucoCEST) MRI with non-contrast injection in predicting the histological grade of rectal cancer.

**Methods:**

This prospective analysis included 60 patients with preoperative rectal cancer who underwent pelvic glucoCEST, amide proton transfer-weighted imaging (APTWI), and diffusion-weighted imaging (DWI). In total, 21 low-grade and 39 high-grade cases were confirmed by postoperative pathology. The MTRasym (1.2 ppm), MTRasym (3.5 ppm), and apparent diffusion coefficient (ADC) values of lesions between the low-grade and high-grade groups were compared. The area under the receiver operating characteristic curve (AUC) was generated to evaluate the diagnostic performance of each technique. Logistic regression (LR) analysis was applied to determine independent predictors and for multi-parameter combined diagnosis.

**Results:**

Elevated MTRasym (1.2 ppm), MTRasym (3.5 ppm) values and lower ADC values were observed in the high-grade group compared with low-grade cases (all *p* < 0.01). The AUCs of MTRasym (1.2 ppm), MTRasym (3.5 ppm), and ADC for differentiating between low- and high-grade rectal cancer cases were 0.792, 0.839, and 0.855, respectively. The diagnostic performance of the combination of the three indexes was improved (AUC, 0.969; sensitivity, 95.24%; specificity, 87.18%). The good consistency and reliability of the combination of independent predictors were demonstrated by calibration curve analysis and DCA.

**Conclusion:**

The glucoCEST MRI without contrast injection, APTWI, and DWI all facilitate the assessment of histological grade in rectal cancer, and the combination of the three can effectively discriminate between high- and low-grade rectal cancer, which is expected to be a promising imaging marker.

**Critical relevance statement:**

The glucose chemical exchange saturation transfer MRI method facilitates the assessment of histological grade in rectal cancer and offers additional information to improve the diagnostic performance of amide proton transfer-weighted imaging, and diffusion-weighted imaging.

**Key Points:**

- Glucose chemical exchange saturation transfer imaging could differentiate histological grade.
- Amide proton transfer-weighted and diffusion-weighted were associated with histological grade.
- The combination of different parameters showed the best diagnostic performance.

**Graphical Abstract:**

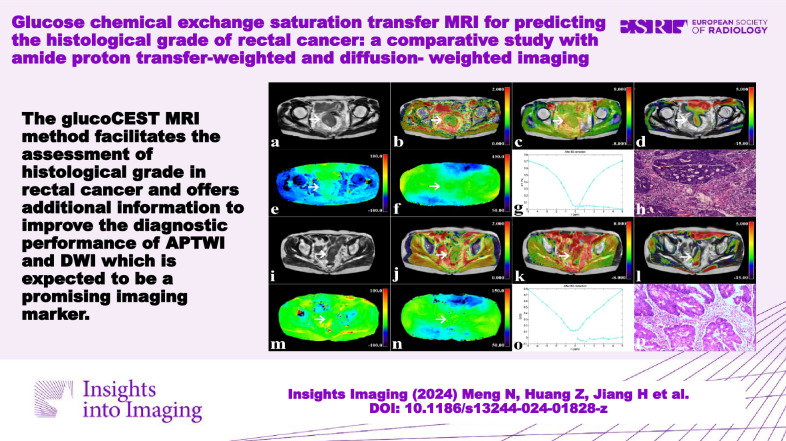

## Introduction

Rectal cancer, one of the deadliest malignancies of the gastrointestinal tract worldwide, is prevalent in individuals over 40 years of age [1]. The histological grade of rectal cancer is an important indicator of aggressiveness level in rectal cancer. This factor has a considerable involvement in treatment strategies and prognosis in relevant patients [2]. Low-grade rectal cancer (intermediate-/highly differentiated), which is confined to the mucosa or submucosa without lymphatic or hematogenous metastasis, can be effectively treated by direct endoscopic resection. However, in high-grade (low-differentiated/undifferentiated) rectal cancer under the same conditions, it is recommended to administer neoadjuvant radiotherapy before surgery [3, 4]. Proctoscopy biopsy is considered the reference standard for determining preoperative grade in rectal cancer [5]. Nonetheless, this approach is invasive and can yield inaccurate histological findings [6]. Therefore, it is of clinical importance to further the development of alternative noninvasive tools for predicting the grade of rectal cancer.

Magnetic resonance imaging (MRI) is commonly utilized for the clinical evaluation of muscular infiltration, lymph nodes and distant metastases of rectal cancer [7], focusing on the macroscopic morphology. However, the histological grade of rectal cancer involves other microscopic lesion features, including cellular morphology, nuclear heterogeneity, etc., which limits the accuracy of conventional MRI in evaluating the grade of rectal cancer [8]. Chemical exchange saturation transfer (CEST) is an emerging molecular MRI technology developed in recent years. CEST has a theoretical principle similar to that of T1rho MRI but is highly specific for certain biochemical components, including proteins [9], glucose [10], and glutamate [11]. Amide proton transfer-weighted imaging (APTWI) is a subtype of the CEST technique that determines the concentrations of mobile macromolecules, including proteins and peptides, which is now widely used for the assessment of various diseases [12]. Previous studies have shown that APTWI can be used to assess histological grade in pelvic tumors such as endometrial carcinomas, cervical cancer, and rectal cancer [13–15]. Glucose chemical exchange saturation transfer (glucoCEST), another subtype of the CEST technique, can detect glucose concentration [16, 17]. A limited number of studies have examined the use of glucoCEST in disease assessment. Xu et al demonstrated the potential use of glucoCEST for the identification of gliomas [18, 19]. In addition, Jin et al found differences in signal intensity derived from glucoCEST between ischemic and normal brain tissues [20]. Furthermore, Walker et al demonstrated that glucoCEST is sensitive to glucose accumulation in colorectal tumors and can differentiate tumors with various pathophysiological and metabolic characteristics [10]. However, most of these studies included only animal models, and, to the best of our knowledge, there is a lack of substantiated evidence to support the use of glucoCEST as a reliable tool for assessing histological grade in rectal cancer patients.

Therefore, the aim of this study was to evaluate the utility of glucoCEST in predicting the histological grade of rectal cancer in clinic. In addition, this study investigated the diagnostic efficacy of glucoCEST versus APTWI and diffusion-weighted imaging (DWI), in order to provide a new reference for relevant clinical studies.

## Materials and methods

### Patients

**Fig. 1:**
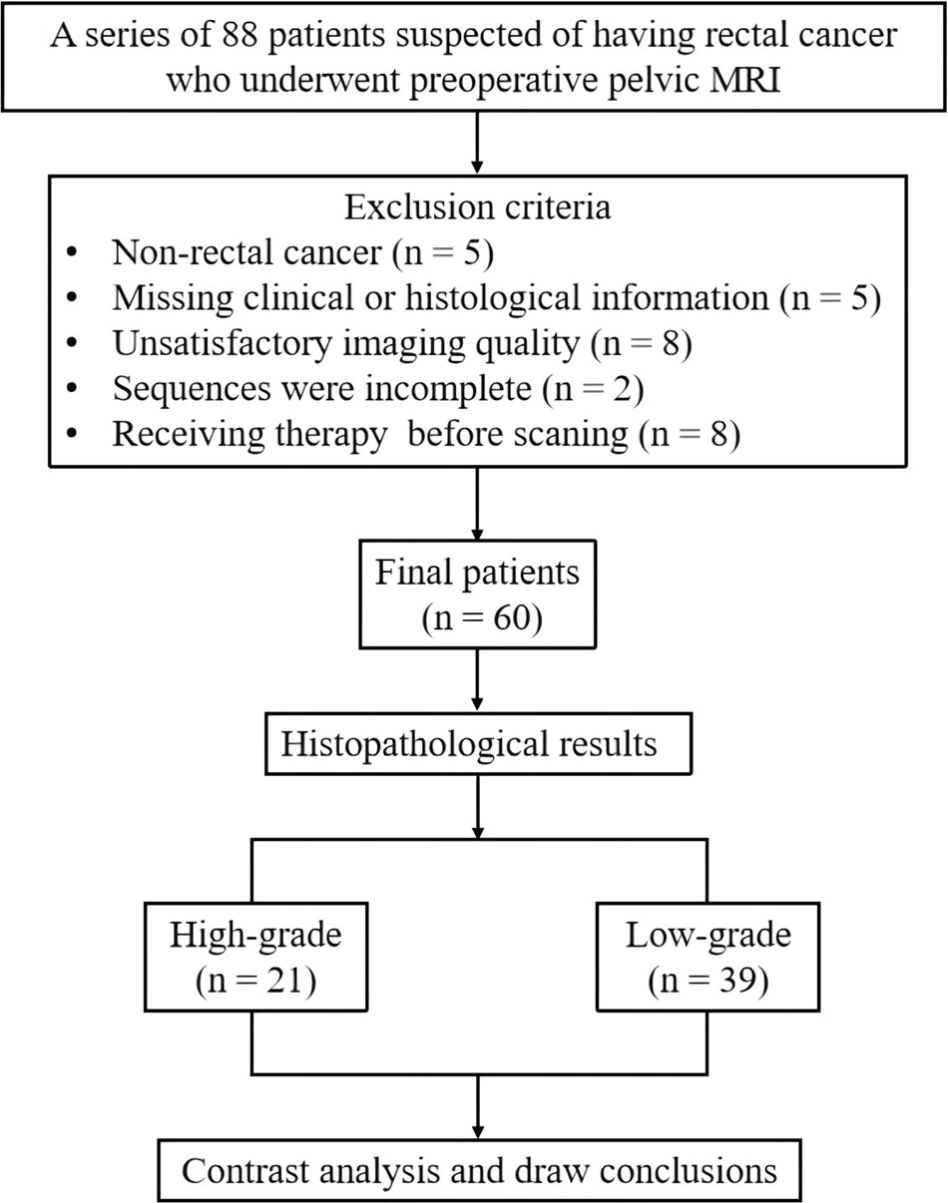
Study flowchart

This prospective study had approval from our hospital’s ethics review committee and each patient provided signed informed consent. From July 2022 to July 2023, 88 individuals with substantial clinical indications of rectal cancer were submitted to pelvic MRI. The following patients were excluded: (1) histological results indicating non-rectal cancer (*n* = 5); (2) missing clinical or histological information (*n* = 5); (3) poor image quality for glucoCEST, APTWI, or DWI (*n* = 8); (4) inability to complete all imaging sequences due to claustrophobia or other physical symptoms (*n* = 2); (5) previous radiotherapy, chemotherapy, or surgery prior to MRI (*n* = 8). Ultimately, a total of 60 patients were included, and clinical data, including gender, age, maximum lesion diameter, classification, location, and tumor stage, were collected. The study flowchart is shown in Fig. 1.

### MRI protocol

MRI was obtained with a 16-channel phased array torso coil on a 3.0-T MR scanner (Ingenia 3.0 T, Philips Healthcare, Best, the Netherlands). Prior to the examination, all patients were instructed to evacuate the rectum and were administered 20 mg of scopolamine butyl bromide (Buscopan; Boehringer) intramuscularly to reduce gastrointestinal movements. Subsequently, each patient was placed supine, feet first, and a respiratory belt was fastened around the abdomen to monitor respiration. DWI (b = 0, 1000 s/mm^2^), APTWI, and glucoCEST were performed, followed by T1-weighted imaging (T1WI), T2-weighted imaging (T2WI), and enhanced T1WI (Magnevist, 0.2 mL/kg body weight, Bayer Healthcare). APTWI was performed with a 3D single-shot TSE-Dixon pulse sequence with inversion recovery (SPIR) fat suppression. The duration of saturation pulse train for the APTWI sequence was 1 s, with a B_1_ root mean square (B_1_, rms) of 1.0 μT. Seven saturation frequency offsets (± 3.5, ± 3.42, ± 3.58, −1560 ppm) were applied to acquire APTWI images. At +3.5 ppm, three images with different echo times (ΔTE = 0.4 ms) were acquired for the asymmetry calculation of B_0_ map applying the mDixon algorithm. GlucoCEST was performed with a 2D single-shot TSE-mDixon sequence with SPIR fat suppression. The saturation frequency and duration of radiofrequency saturation pulse train for the glucoCEST sequence were 1.2 ppm and 1 s, respectively, and B_1_ root mean square (B_1_, rms) was 2.0 μT. To acquire a z-spectrum, imaging was repeated at 20 saturation frequency offsets of −5, −3.5, −3, −2, −1.5, −1.2, −0.8, −0.5, −0.25, 0, 0.25, 0.5, 0.8, 1.2, 1.5, 2, 3, 3.5, 5 ppm, as well as one offset far from the resonant frequency (1024.0 ppm). The protocol’s details are provided in Table 1.

**Table 1:** Imaging protocol parameters

Parameters	T1WI	T2WI	DWI	APTWI	glucoCEST	Enhanced T1WI
Sequence	2D-TSE	2D-TSE	2D-EPI	3D-TSE	2D-TSE	DIXON
Orientation	Axial	Axial	Axial	Axial	Axial	Axial
Flip angle (degree)	90	90	90	90	90	10
TR/TE (ms)	550/8	5108/125	6000/54	10,000/7.8	6800/7.7	3.8/1.3
FOV (cm^2^)	30 × 38	30 × 38	35 × 50	35 × 50	35 × 50	36 × 31
Matrix	379 × 339	300 × 250	132 × 128	120 × 120	120 × 120	240 × 200
Slice thickness (mm)	6	6	6	6	1	4
Interval (mm)	1	1	1	1	0	−2
NEX	1.5	2	1	1	1	1
ETL	8	18	–	–	–	–
Fat suppression	No	No	SPIR	SPIR	SPIR	No
b-values (s/mm^2^)	–	–	1000	–	–	–
Respiratory compensation	Free	Free	Free	Free	Free	Breath Hold
Scan time	1 min 04 s	1 min 24 s	1 min 48 s	1 min 30 s	2 min 30 s	18 s

*TSE* turbo spin echo, *EPI* echo planar imaging, *TR/TE* repetition time/echo time, *FOV* field of view, *NEX* number of excitations, *ETL* echo train length, *SPIR* spectral presaturation with inversion recovery

### Parameter generation

All images were uploaded to the post-processing Intellispace Portal Workstation (ISP, version 9, Philips Healthcare, the Netherlands), to generate the ADC map with the MR diffusion software package. B_0_/B_1_ mapping and B_0_-corrected 3D APTWI images were auto-constructed on the MR scanner. 2D-glucoCEST images were post-processed with a custom-made program in MATLAB software for the acquired z-spectra and MTRasym (1.2 ppm) mapping [21]. The DWI-derived parameter apparent diffusion coefficient (ADC) was calculated using Eq. 1:

1 $${S}_{{{{\mathrm{b}}}}}={{{{\mathrm{S}}}}}_{0}\cdot \exp (-{{{\rm{b}}}}\cdot ADC)$$

where b represents the diffusion sensitizing factor; S_0_ and S_b_ represent signal intensities (SIs) at a b-value of 0 and the b-value indicated by the subscript, respectively. The glucoCEST- and APTWI-derived parameters magnetization transfer ratio asymmetry at 1.2 ppm downfield from the water signal (MTRasym (1.2 ppm)) and magnetization transfer ratio asymmetry at 3.5 ppm downfield from the water signal (MTRasym (3.5 ppm)) were derived from Eqs. 2 and 3, respectively:

2 $$MTR{{{\rm{asym}}}}(3.5\,ppm)=[{S}_{sat}(-3.5\,ppm)-{S}_{sat}(+3.5\,ppm)]/{S}_{0}$$

3 $$MTR{{{\rm{asym}}}}(1.2\;ppm)=[{S}_{sat}(-1.2\;ppm)-{S}_{sat}(+1.2\;ppm)]/{S}_{0}$$

where S_0_ and S_sat_ are signal intensities (SIs) obtained without and with selective saturation, respectively. The regions of interest (ROIs) were manually placed along the mass tumor area at the slice of the largest area on axial DWI images with reference to T2WI and enhanced T1WI images. Areas with cystic degeneration, necrosis, apparent signs, hemorrhage artifacts and blood vessels were avoided. Then, the ROIs were copied to the ADC, MTRasym (1.2 ppm), and MTRasym (3.5 ppm) pseudo-color maps, and mean ADC, MTRasym (1.2 ppm), and MTRasym (3.5 ppm) values of the ROIs were automatically determined. All the above procedures were independently performed by an attending radiologist and an associate chief radiologist with 8 and 15 years of experience in pelvic MRI, respectively, who were blinded to each other’s results and to the clinicopathological data of patients.

### Histopathologic analysis

All specimens were obtained by surgery within 2 weeks of the MRI examination and sent to the pathology department of our hospital for analysis by a pathologist with 10 years of experience in gastrointestinal pathologic diagnosis who was blinded to imaging data. Hematoxylin-eosin (H&E) staining was performed to determine histological features. Referring to gland or tubule formation and architecture in the lesion, each rectal cancer specimen was categorized as grade 1 (well-differentiated, > 95% gland formation), grade 2 (moderately differentiated, 50–95% gland formation), and grade 3 (poorly differentiated, < 50% gland formation) [22]. Grade 1 and 2 samples were assigned to the low-grade group, while grade 3 cases were in the high-grade group.

### Statistical methods

R (version 3.5.3; R Foundation, Auckland, New Zealand) and SPSS (version 15.0; MedCalc Software, Ostend, Belgium) were used for data analysis. Interobserver agreement between the two radiologists for glucoCEST SIs, MTRasym (3.5 ppm), and ADC values was assessed using the intra/interclass correlation coefficient (ICC), with an ICC > 0.75 indicating adequate reliability [13]. Between the high- and low-grade rectal cancer groups, categorical variables were compared by the chi-square test, while continuous variables were compared by the Mann–Whitney *U* test or independent samples *t*-test. The diagnostic efficacy was evaluated using the area under the receiver operating characteristic curve (AUC), and AUCs were compared by the DeLong test. Logistic regression (LR) analysis was employed to identify independent predictors and generate a multi-parameter composite diagnostic tool. A control model was built by bootstrapping (1000 samples), and its performance was assessed by calibration curve analysis, decision curve analysis (DCA), and ROC curve analysis. Statistical significance was set at *p* < 0.05.

## Results

### Baseline patient data

A total of 21 high-grade and 39 low-grade rectal cancer cases were enrolled in this study. There were significant differences in gender and maximum lesion diameter between the high- and low-graded groups (*p* = 0.047 and *p* = 0.003) and no significant differences in age, classification, location, and tumor stage (all *p* > 0.05). The above clinical characteristics are presented in Table 2.

**Table 2:** Comparison of pathologic characteristics and different parameters

Variable	High-grade (*n* = 21)	Low-grade (*n* = 39)	t/z /χ^2^ value	*p*-value
Age (years)	59.67 ± 12.81	62.33 ± 9.58	0.836	0.409^a^
Maximum diameter (mm)	43.62 ± 14.24	32.26 ± 11.36	−3.156	0.003^a^
Sex, *n* (%)			3.956	0.047^b^
Male	9 (42.86%)	27 (69.23%)		
Female	12 (58.14%)	12 (30.77%)		
TNM stage, *n* (%)			2.475	0.480^b^
I	6 (28.58%)	18 (46.15%)		
II	2 (9.52%)	5 (12.82%)		
III	11 (52.38%)	13 (33.33%)		
IV	2 (9.52%)	3 (7.69%)		
Classification, *n* (%)			1.471	0.689^b^
Ulcerative	8 (38.09%)	12 (30.77%)		
Bulge	8 (38.09%)	21 (53.85%)		
Infiltrating	4 (19.05%)	5 (12.82%)		
Ulcerative eminence	1 (4.76%)	1 (2.56%)		
Location, *n* (%)			1.105	0.575^b^
Low	10 (47.62%)	15 (38.46%)		
Median	8 (38.10%)	14 (35.90%)		
High	3 (14.28%)	10 (25.64%)		
Parameters				
ADC (10^−^^3^ mm^2^/s)	0.95 ± 0.19	1.32 ± 0.32	4.951	< 0.001^a^
MTRasym (3.5 ppm) (%)	1.88 (1.29, 5.45)	0.83 (0.37, 1.46)	−4.301	< 0.001^c^
MTRasym (1.2 ppm) (%)	2.20 (−1.76, 7.41)	−3.23 (−8.14, 0.61)	−3.712	< 0.001^c^
B_0_ (Hz)	11.98 (−2.25, 37.15)	9.89 (−1.94, 32.48)	−0.101	0.920^c^
B_1_ (%)	90.46 ± 14.24	84.91 ± 14.78	−1.416	0.163^a^

*MTRasym (1.2 ppm)* magnetization transfer ratio asymmetry at 1.2 ppm, *ADC* apparent diffusion coefficient, *MTRasym (3.5 ppm)* magnetization transfer ratio asymmetry at 3.5 ppm

^a^ Independent samples *t*-test

^b^ Chi-square test

^c^ Mann–Whitney *U* test

### Data consistency

The interclass correlation coefficients of ADC, MTRasym (3.5 ppm) and MTRasym (1.2 ppm) values were 0.939 (95% CI: 0.898–0.964), 0.952 (95% CI: 0.919–0.971), and 0.976 (95% CI: 0.960–0.986), respectively. The intraclass correlation coefficient of ADC, MTRasym (3.5 ppm) and MTRasym (1.2 ppm) values were 0.919 (95% CI: 0.849–0.955), 0.931 (95% CI: 0.885–0.959), and 0.949 (95% CI: 0.915–0.970), respectively. The average results were used for further analysis.

### Image parameters

**Fig. 2:**
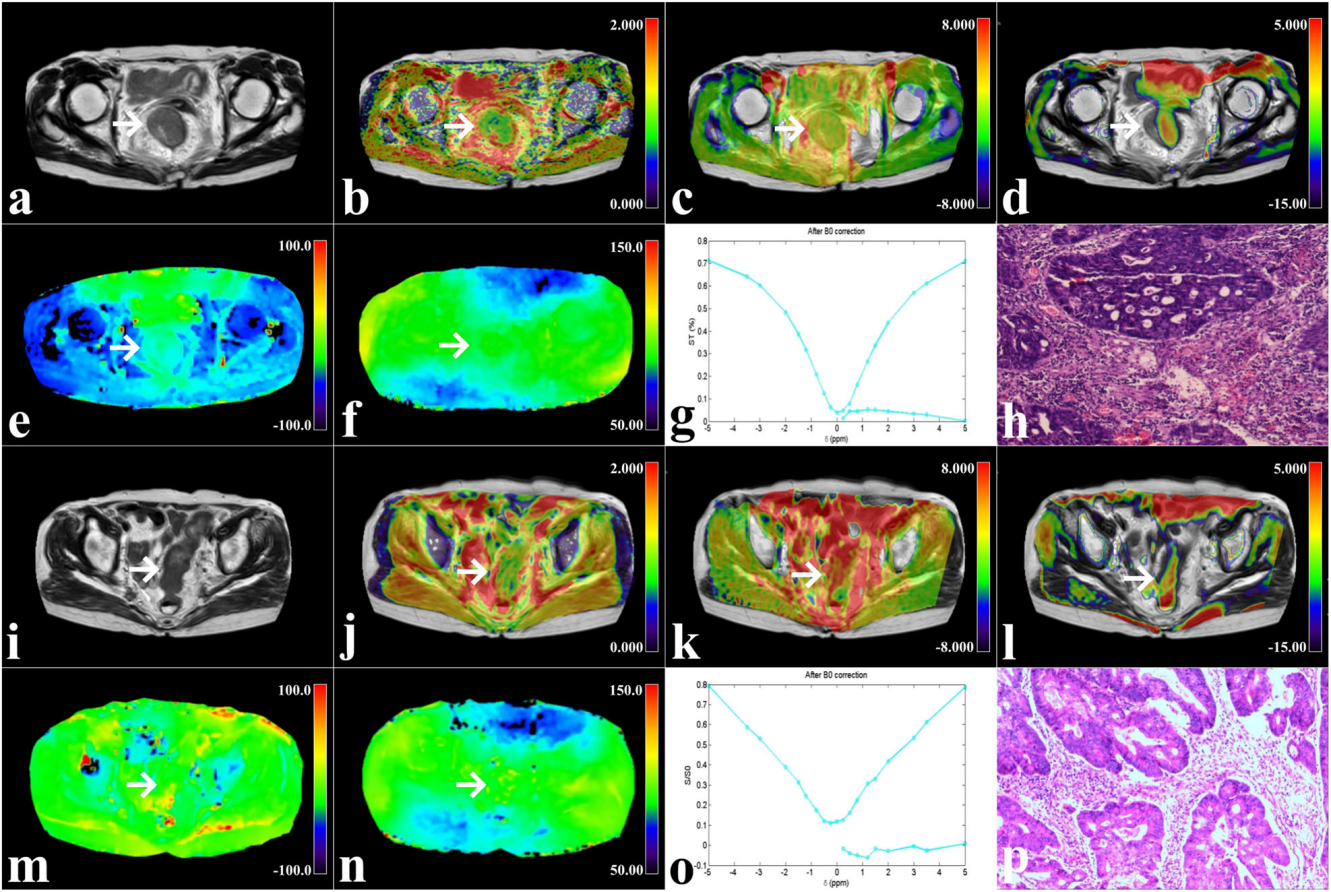
**a**–**h** A 76-year-old woman with rectal cancer (arrowheads, maximum diameter 45 mm, Grade 3). **i**–**p** A 70-year-old woman with rectal cancer (arrowheads, maximum diameter 42 mm, Grade 2). **a**, **i** T2WI maps. **b**, **j** Fusion images of ADC pseudo-color and T2WI maps. **c**, **k** Fusion images of MTRasym (3.5 ppm) pseudo-color and T2WI maps. **d**, **l** Fusion images of MTRasym (1.2 ppm) pseudo-color and T2WI maps. **e**, **m** B_0_ maps of glucoCEST. **f**, **n** B_1_ maps of glucoCEST. **g**, **o** Z-spectra of glucoCEST. **h**, **p** Pathology images (H&E staining, 100×)

**Fig. 3:**
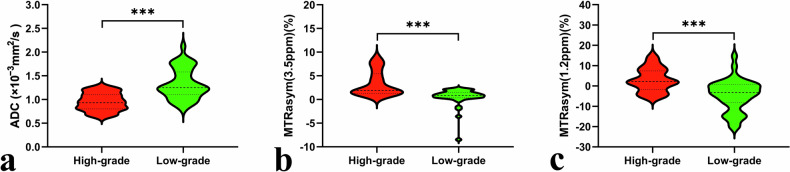
Violin plots show individual data points, averages, and standard deviations of ADC (**a**), MTRasym (3.5 ppm) (**b**), and MTRasym (1.2 ppm) (**c**) in high- and low-grade rectal cancer. ***p < 0.001

ADC was significantly lower and MTRasym (3.5 ppm), MTRasym (1.2 ppm) were significantly higher in the high-grade group compared with the low-grade group (all *p* < 0.001). Figures 2 and 3 and Table 2 summarize image parameters for each sequence.

### Risk factors

Possible risk factors, including age, maximum lesion diameter, gender, tumor stage, ADC, MTRasym (3.5 ppm), and MTRasym (1.2 ppm), were all included in the LR analysis. Univariate analysis demonstrated that gender, maximum lesion diameter, ADC, MTRasym (3.5 ppm), and MTRasym (1.2 ppm) were all predictors of rectal cancer grade (all *p* < 0.1), while multivariate analysis demonstrated that only ADC, MTRasym (3.5 ppm), and MTRasym (1.2 ppm) were independent predictors (*p* = 0.018, 0.040, and 0.042, respectively, Table 3).

**Table 3:** Univariate and multivariate analyses

Variables	Univariate analyses		Multivariate analyses	
	OR (95% CI)	*p*-value	OR (95% CI)	*p*-value
Age (year)	0.977 (0.930–1.027)	0.362	–	–
Sex	0.333 (0.111–1.001)	0.050	0.355 (0.044–2.811)	0.332
Maximum diameter (mm)	1.078 (1.024–1.136)	0.004	0.997 (0.907–1.096)	0.954
Stage	1.448 (0.863–2.431)	0.161	–	–
Classification	1.104 (0.541–2.254)	0.785	–	–
Location	0.701 (0.344–1.428)	0.328	–	–
ADC (10^−^^3^ mm^2^/s)	0.001 (0.000–0.054)	< 0.001	0.001 (0.000–0.281)	0.018
MTRasym (3.5 ppm) (%)	3.759 (1.505–9.388)	0.005	3.614 (1.063–12.280)	0.040
MTRasym (1.2 ppm) (%)	1.187 (1.065–1.323)	0.002	1.221 (1.008–1.480)	0.042

Both univariate and multivariate analyses were conducted using the forward LR method

*MTRasym (1.2 ppm)* magnetization transfer ratio asymmetry at 1.2 ppm, *ADC* apparent diffusion coefficient, *MTRasym (3.5 ppm)* magnetization transfer ratio asymmetry at 3.5 ppm, *OR* odds ratio, *CI* confidence interval

### Diagnostic performance

**Fig. 4:**
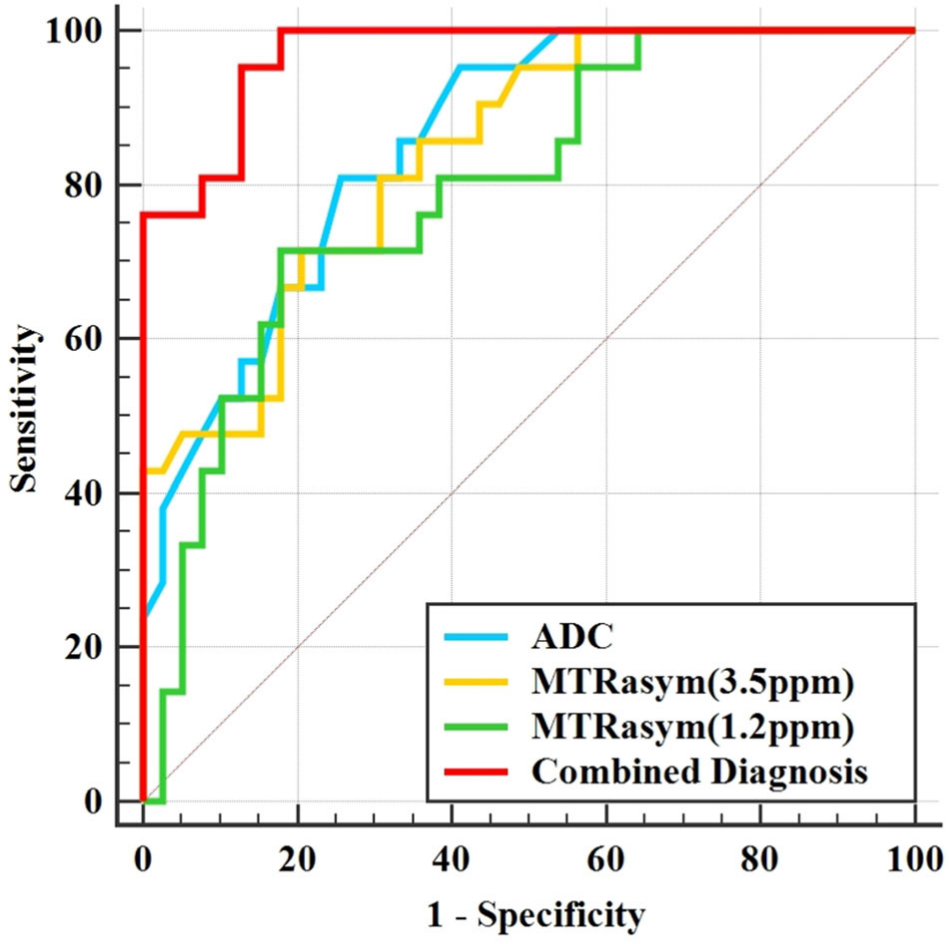
The areas under receiver-operator characteristic (ROC) curves of different parameters and the combination of independent predictors (ADC + MTRasym (3.5 ppm) + MTRasym (1.2 ppm))

For differentiating between high- and low-grade rectal cancer cases, the combination of independent predictors (ADC, MTRasym (3.5 ppm), and MTRasym (1.2 ppm)) showed the best diagnostic performance (AUC = 0.969; sensitivity, 95.24%; specificity, 87.18%), surpassing ADC, MTRasym (3.5 ppm), and MTRasym (1.2 ppm) with statistical significance (AUCs = 0.855, 0.839, and 0.792, respectively; Z = 2.737, 2.788, and 2.991, respectively, *p* = 0.006, 0.005, and 0.003, respectively). There were no statistically significant differences in AUC between MTRasym (3.5 ppm) and MTRasym (1.2 ppm), and ADC (Z = 0.613, 0.212, *p* = 0.540, 0.832, respectively), or between MTRasym (1.2 ppm) and ADC (Z = 0.818, *p* = 0.413; Fig. 4 and Table 4).

**Table 4:** Predictive performance for identifying benign and malignant SPL

Parameters	AUC (95% CI)	*p*-value	Cutoff	Sensitivity	Specificity	Comparison with a combined diagnosis
ADC (10^−^^3^ mm^2^/s)	0.855 (0.740–0.932)	< 0.001	1.100	80.95%	74.36%	Z = 2.737, *p* = 0.006
MTRasym (3.5 ppm) (%)	0.839 (0.721–0.921)	< 0.001	1.550	71.43%	79.49%	Z = 2.788, *p* = 0.005
MTRasym (1.2 ppm) (%)	0.792 (0.668–0.886)	< 0.001	0.810	71.43%	82.05%	Z = 2.991, *p* = 0.003
Combined diagnosis	0.969 (0.889–0.997)	< 0.001	–	95.24%	87.18%	–

Combined diagnosis represents ADC + MTRasym (3.5 ppm) + glucoCEST SIs

*ADC* apparent diffusion coefficient, *MTRasym (3.5 ppm)* magnetization transfer ratio asymmetry at 3.5 ppm, *MTRasym (1.2 ppm)* magnetization transfer ratio asymmetry at 1.2 ppm

### Validation

**Fig. 5:**
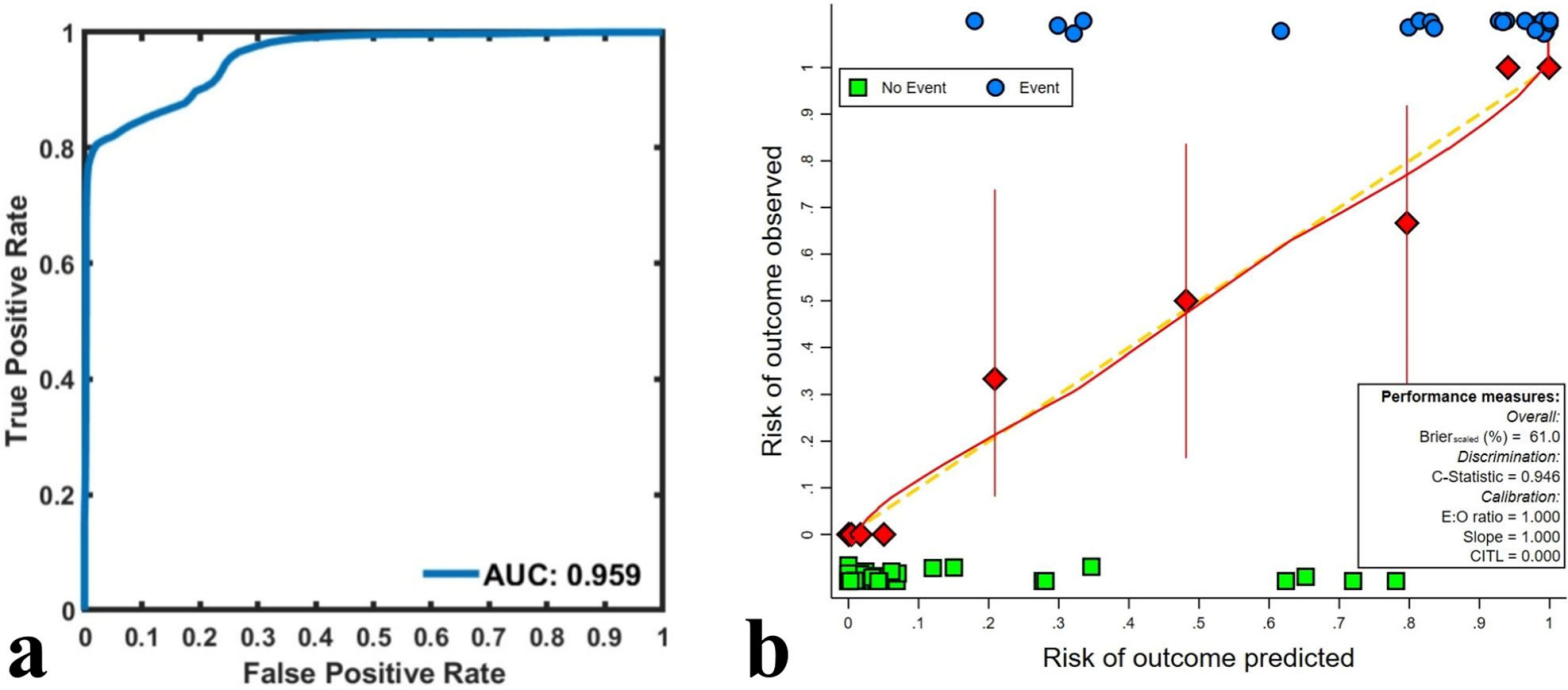
ROC curve (**a**) and calibration curve (**b**) analyses of the validation model

**Fig. 6:**
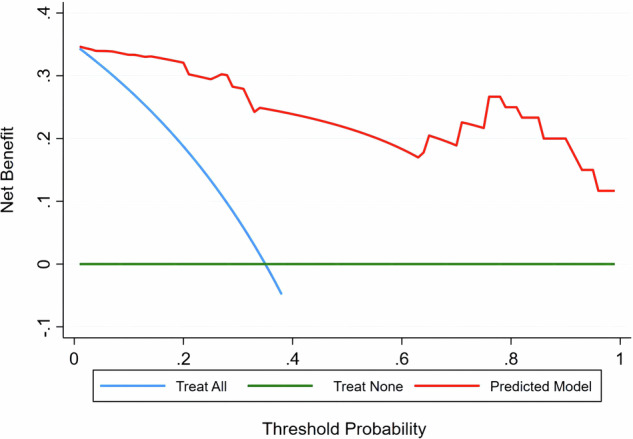
Decision curve analysis of the combination of independent predictors (ADC + MTRasym (3.5 ppm) + MTRasym (1.2 ppm))

In the control model based on bootstrapped samples, the combination of independent predictors also had a good predictive value, with an AUC of 0.959 (95% CI: 0.922–0.969, Fig. 5a). Calibration and DCA curves revealed that the combination of independent predictors exhibited not only good consistency but also provided reliable clinical benefits to rectal cancer patients (Figs. 5b, 6).

## Discussion

An accurate preoperative assessment of histological grade in rectal cancer is essential due to different clinical management tools and prognoses in high- and low-grade cancers. This study investigated the potential of DWI, APTWI, and glucoCEST based on MRI for distinguishing between high- and low-grade rectal cancer. Our findings revealed that high-grade rectal cancer cases had elevated MTRasym (1.2 ppm) and MTRasym (3.5 ppm), and decreased ADC values in comparison with the low-grade rectal cancer group. In addition, ADC, MTRasym (3.5 ppm), and MTRasym (1.2 ppm) had good diagnostic values in differentiating between high- and low-grade rectal cancers, with AUCs of 0.855, 0.839, and 0.792, respectively. Furthermore, when these parameters were combined, a higher diagnostic performance was obtained compared with each parameter individually.

DWI is the most widely used quantitative MRI sequence in clinical practice, and its derived parameter ADC effectively reflects the degree of restricted diffusion movement of water molecules in biological tissues [23]. Previous studies have reported that high-grade rectal cancers show significantly lower ADC values compared with low-grade rectal cancers due to increased cell proliferation and denser tissue structure, suggesting the more limited diffusion of water molecules within the tumor [24, 25]. This study corroborated the above studies and further found that ADC served as an independent index for differentiating between high- and low-grade rectal cancer cases, which provides additional evidence supporting the application of DWI in the assessment of histological grade in rectal cancer.

APTWI is a subtype of the CEST imaging sequence. It facilitates the detection of variations in protein and peptide concentrations in biological tissues to water molecules using hydrogen proton exchange and quantifies this alteration through the derived parameter MTRasym (3.5 ppm) [9]. Nishie et al examined the application of APTWI in predicting pathological factors of rectal cancer, suggesting that MTRasym (3.5 ppm) can be used to differentiate between high- and low-grade rectal cancers [15]. Takayama et al reported that MTRasym (3.5 ppm) in endometrioid endometrial adenocarcinoma is positively correlated with histological grade [13]. Moreover, Wang et al also showed a potential for APTWI in contributing to the evaluation of histological grade in bladder cancer [26]. The current results showed that MTRasym (3.5 ppm) was higher in the high-grade rectal cancer group compared with the low-grade rectal cancer group, corroborating the above studies. A possible explanation is that compared with low-grade cancers, high-grade cancers are characterized by faster cell proliferation, higher cell density, and increased protein expression, and therefore have higher concentrations of mobile proteins and peptides [27]. In addition, nuclear atypia and microscopic necrosis may also be attributed to higher MTRasym (3.5 ppm) in high-grade cancer [13, 28].

Besides APTWI, glucoCEST is another subtype of the CEST technique, which is based on proton exchange between hydroxyl and water molecules and enables noninvasive quantitative assessment of glucose concentration in tissues [29]. Using glucoCEST, Xu et al scanned mice orthotopically injected with human glioma cells and found elevated glucoCEST signals in tumor tissues compared with normal brain tissues [18]. Walker et al assessed two mouse xenograft models of subcutaneous human colorectal tumor using both ^18^F-FDG PET and glucoCEST. Their results indicated a positive correlation between ^18^F-FDG PET and glucoCEST signals in different tumor subtypes. Moreover, a significant level of agreement was observed between both methods in the assessment of glucose metabolism [10]. In this study, we first applied glucoCEST to determine the histological grade in rectal cancer cases, and a significant increase in MTRasym (1.2 ppm) was found in the high-grade rectal cancer group compared with low-grade rectal cancer cases. This finding might be attributed to higher hydroxyl concentrations in high-grade tumors, which are characterized by faster cell proliferation, increased glucose metabolism, and more significant hypoxia [30, 31]. The increased T1 relaxation time may also enhance the signal intensity of glucoCEST [32]. Li et al reported that T1 relaxation time was increased in the high-grade rectal cancer group compared with low-grade rectal cancer cases [33]. Therefore, we speculate that the difference in T1 relaxation time might also contribute to the variation of MTRasym (1.2 ppm) between high- and low-grade rectal cancers in this study.

Several reports have shown that injecting D-glucose into animals before scanning enhances the glucoCEST signal, and thus improves the quantitative accuracy of glucoCEST [18, 34]. Furthermore, studies have shown that satisfactory glucoCEST imaging is possible without the administration of D-glucose [35]. The accurate determination of the time-signal characteristics of body organs, including the rectum, subsequent to glucose intake might be challenging, and some patients may be unable to receive D-glucose injection due to medical conditions such as diabetes mellitus. Therefore, the use of glucoCEST imaging in rectal cancer patients without D-glucose injection was investigated in this study. The results revealed that glucoCEST not only could display rectal cancer lesions but also yielded a stable Z-spectrum effect, which suggests glucoCEST can be performed without D-glucose injection in rectal cancer patients. However, compared with the APTWI-derived parameter MTRasym (3.5 ppm), the glucoCEST-derived parameter MTRasym (1.2 ppm) is closer to the water peak and more susceptible to B_0_ inhomogeneity and B_1_ inhomogeneity [36]. In this study, the WASSR-based technique was utilized to automatically generate B_0_ maps and correct for B_0_ inhomogeneity. In addition, to assess the impacts of lesion B_0_ and B_1_, separate B_0_ and B_1_ maps were obtained, and B_0_ and B_1_ values were measured at the lesions in this study; as a result, there were no statistically significant differences in B_0_ and B_1_ values between high-grade and low-grade rectal cancers. These results provide convincing evidence that MTRasym (1.2 ppm) measured in this study has a high degree of stability.

The present study revealed the presence of negative MTRasym (1.2 ppm) values for some lesions in both the high-grade and low-grade rectal groups. These negative values were more abundant in the low-grade group compared with the high-grade group. We speculate that this may be a result of reduced glucose content within some lesion tissues [37]. Furthermore, because of the anatomical characteristics of the rectum as a hollow organ, it is possible for gas, feces, or fluid to remain in the bowel even after the patient has been advised to evacuate the rectum prior to scanning. The presence of residual substances within the bowel can potentially contribute to the occurrence of negative values in this study. In addition, it can be challenging to completely avoid these areas when delineating the ROIs. Furthermore, several challenges in glucoCEST body imaging may also explain the above results [38].

Although this study yielded encouraging results, it is important to acknowledge several limitations. First, this study was conducted at a single institution, and the sample size was relatively small, which might affect the reliability of the results. Second, due to the low spatial resolution of glucoCEST and APTWI, some small lesions were excluded, which might introduce sampling bias. Third, ROIs were delineated only in the largest area of the lesion rather than considering the entire lesion, which may also result in some degree of bias. Fourth, 2D-glucoCEST imaging required a long time, likely leading to discomfort and potential motion interference in some individuals, particularly the elderly. Fifth, the imaging and post-processing methods used for glucoCEST and APTWI in this study are still in the early stages of development [39, 40]. To enhance imaging quality and effectively isolate pure hydroxyl/amide proton transfer signals from the influence of other components, e.g., the nuclear Overhauser-mediated CEST effect, it is essential for future studies to address certain technical challenges.

## Conclusion

The glucoCEST MRI without contrast injection, APTWI, and DWI all facilitate the assessment of histological grade in rectal cancer, and the combination of the three can effectively discriminate between high- and low-grade rectal cancer, which is expected to be a promising imaging marker.

## Data Availability

All data are with the corresponding author and can be obtained by mail if necessary.

## Abbreviations

ADC: Apparent diffusion coefficient
APTWI: Amide proton transfer-weighted imaging
AUC: Area under the receiver operating characteristic curve
CEST: Chemical exchange saturation transfer
DWI: Diffusion-weighted imaging
GlucoCEST: Glucose chemical exchange saturation transfer
LR: Logistic regression
MRI: Magnetic resonance imaging
MTRasym (1.2 ppm): Magnetization transfer ratio asymmetry at 1.2 ppm
MTRasym (3.5 ppm): Magnetization transfer ratio asymmetry at 3.5 ppm
